# Understanding Plant Nitrogen Metabolism through Metabolomics and Computational Approaches

**DOI:** 10.3390/plants5040039

**Published:** 2016-10-10

**Authors:** Perrin H. Beatty, Matthias S. Klein, Jeffrey J. Fischer, Ian A. Lewis, Douglas G. Muench, Allen G. Good

**Affiliations:** 1 Department of Biological Sciences, University of Alberta, 85 Avenue NW, Edmonton, AB T6G 2E9, Canada; allen.good@ualberta.ca; 2 Department of Biological Sciences, University of Calgary, 2500 University Drive NW, Calgary, AB T2N 1N4, Canada; mklein@ucalgary.ca (M.S.K.); jfischer@roevin.ca (J.J.F.); ian.lewis2@ucalgary.ca (I.A.L.); dmuench@ucalgary.ca (D.G.M.)

**Keywords:** metabolomics, nitrogen, nitrogen use efficiency (NUE), transgenic crops, nitrogen uptake efficiency (NUpE), nitrogen utilization efficiency (NUtE), flux balance analysis (FBA), N boundary, mass spectrometry (MS), nuclear magnetic resonance (NMR)

## Abstract

A comprehensive understanding of plant metabolism could provide a direct mechanism for improving nitrogen use efficiency (NUE) in crops. One of the major barriers to achieving this outcome is our poor understanding of the complex metabolic networks, physiological factors, and signaling mechanisms that affect NUE in agricultural settings. However, an exciting collection of computational and experimental approaches has begun to elucidate whole-plant nitrogen usage and provides an avenue for connecting nitrogen-related phenotypes to genes. Herein, we describe how metabolomics, computational models of metabolism, and flux balance analysis have been harnessed to advance our understanding of plant nitrogen metabolism. We introduce a model describing the complex flow of nitrogen through crops in a real-world agricultural setting and describe how experimental metabolomics data, such as isotope labeling rates and analyses of nutrient uptake, can be used to refine these models. In summary, the metabolomics/computational approach offers an exciting mechanism for understanding NUE that may ultimately lead to more effective crop management and engineered plants with higher yields.

## 1. Introduction

Nitrogen (N) is a significant contributor to plant biomass and an essential component of most biomolecules. Nitrogen limitation frequently reduces crop growth and yield, and contributes to a variety of phenotypic changes including: expanded root architecture, reduced shoot biomass production, chlorosis, leaf discoloration, and impaired reproduction [1]. To ensure sufficient N for crop growth, farmers supplement fields with fertilizers containing nitrate (NO_3_^−^), ammonium (NH_4_^+^), or urea (CO(NH_2_)_2_). Most crops take up approximately 40% of applied N [2], which results in environmental pollution in the form of aerosolized nitrous oxides and leaching of soluble nitrates into waterways [3,4,5]. Improving the nitrogen use efficiency (NUE) of crops might allow existing agricultural technologies to decrease the yield gap and therefore increase the necessary caloric intake of billions of people worldwide, and could diminish the impact of agriculture on the environment.

Efforts are currently underway to improve NUE through a variety of strategies. These include a critical evaluation of the method, timing, and management of fertilizer use as well as strategies for crop rotation and legislative changes to promote best management practices [2,6]. For example, Denmark now requires liquid fertilizers to be injected directly into the soil, which can reduce nitrogen volatilization by 41% [7]. In addition to these legislative actions, scientific investigations are ongoing to improve NUE though crop genetics. These efforts have included both traditional breeding programs and modern recombinant DNA technology (reviewed in [1,8,9,10]). One of these molecular approaches has resulted in crops achieving a higher NUE via the overexpression of alanine aminotransferase [8].

The success of modern molecular genetics has stimulated interest in adapting other state-of-the-art scientific strategies to this traditional agricultural problem. One advanced scientific approach with relevance to NUE is metabolomics. Many aspects of cellular metabolism have a direct impact on the uptake of nitrogen and partitioning of this element in tissues [11,12]. Given the significant resources required to develop transgenic crops [13,14], it would be prudent to implement a technology that can directly evaluate the relationships between genetic change and nitrogen utilization. Decades of research have established detailed metabolic profiles of many crops [15], profiling the transcriptional response of N metabolism-related genes to diverse stimuli [16,17,18,19], and mapping of quantitative crop phenotypes to loci associated with metabolic genes [20,21,22]. Despite this wealth of information, the fundamental metabolic limitations of NUE remain unclear.

One major challenge in understanding NUE is the staggering scale of the metabolic networks of plants. There are thought to be more than 10,000 metabolites present in plants [23,24]. In addition, the metabolic activity of organisms is established through the combined action of numerous genes, chemical equilibria, and multiple layers of regulation. Consequently, unravelling the complex interactions between NUE and metabolism requires analytical approaches that can capture a comprehensive picture of steady-state metabolite concentrations and metabolic pathway fluxes.

Although extensive data is available on the metabolism of crop species [1,25], traditional analytical limitations have restricted metabolic investigations to a handful of metabolites per study. However, recent advances in analytical technology have dramatically increased the potential scope of metabolism research and have made comprehensive analyses of plant networks a feasible objective [26,27,28,29,30]. These metabolomics studies allow comprehensive data to be captured on the abundance of metabolites, activities of metabolic pathways, and physiological partitioning of nutrients. Herein, we describe recent advances in NUE research and discuss how metabolomics techniques could be harnessed to improve our understanding of NUE in crops.

## 2. Discussion

### 2.1. Role of Metabolism in NUE

Plant nitrogen uptake, assimilation and metabolism have been studied for over a century in regards to growth and yield of agriculturally important plants [8,31]. Perturbations in this essential physiological process have significant impact on the phenotypes of plants and elicit major changes in their metabolic networks. Nitrogen metabolism has been extensively examined in the context of N starvation ([11,26,32,33,34]; summarized in Table 1). Metabolic changes in plants are affected by length of the starvation period [23,24], tissue type [23], whether or not the plant is a cultivated variety [26,35], and developmental stage [11]. Not surprisingly, N starvation generally results in diminished levels of nitrogen containing metabolites (Table 1); this is most evident in normally abundant amino acids such as glutamate and glutamine. Depletion of these amino acids is generally correlated with elevated levels of organic acids from central carbon metabolism (Table 1). The metabolic composition is closely correlated, and tightly regulated, with plant biomass so that the metabolic composition can be a predictor (biomarker) of biomass [36]. Certain metabolites; carbon compounds from photosynthesis, starch and sucrose metabolism plus oxidative pentose phosphate pathway, tricarboxylic acid (TCA) cycle and glycolysis metabolites and N-containing metabolites such as glutamine, have been seen to be at low levels in plants undergoing high growth. This suggests that these metabolites are providing the major building blocks for biomass macromolecules such as proteins and that growth drives metabolism [36]. The majority of the plant’s nitrogen stores are held as protein biomass or as inorganic nitrogen in the vacuoles. Consequently, the most effective NUE engineering strategies will likely target the flow of C and N through the metabolic network rather than focusing on concentrations of individual metabolites. Another theory regarding metabolites and growth suggests that the levels of some metabolites act as signaling molecules and regulate plant growth either positively or negatively. There are many metabolites of unknown structure that may be derived from primary metabolites to act as signaling molecules that should be studied further to understand this metabolite–growth interaction [36].

### 2.2. Effect of Transgene Expression on Nitrogen Metabolism

A common strategy for attempting to improve NUE has been to genetically modify plants [8,9,10]. Efforts have focused on (1) transgenes targeting N uptake and transport, such as ammonium transporters [37], proton gradient-forming ATPases [38], or peptide/nitrate transporters [39]; (2) transgenes directly involved in primary N metabolism such as cytosolic glutamine synthetase (GS1;1 and GS1;2) [40,41,42], plastidic glutamine synthetase (GS2) [43], glutamate synthase (GOGAT) [44], glutamate dehydrogenase (GDH, [45] or primary and secondary N metabolism such as transaminases like asparagine synthetase (AS1) [46] and alanine aminotransferase (AlaAT) [12]; (3) transgenes involved in N recycling such as autophagy-related factor 8c (ATG8c, [47]); (4) regulatory factors such as the transcription factor Dof1 [48,49], microRNA826 [50], or microRNA444 [51]; (5) or N-responsive transgenes of unknown function such as the early nodulin 93-like gene [52]. Although there have not been any detailed metabolomics studies involving plants with genetically engineered NUE phenotypes, several studies have examined specific metabolite levels, some of these studies are listed in Table 2 [12,41,46,48,49,52,53,54]). The studies in Table 2 demonstrated that over-expression of transgenes involved in nitrogen metabolism altered the levels of primary nitrogen compounds in certain tissues such as roots and shoots. However, in many of these studies, alteration of N metabolite levels either did not result in a subsequent NUE phenotype, or the potential NUE phenotype was not investigated. For example, GS1;1 and GS1;2 overexpression in rice (separately) increased core C and N metabolites in roots and shoots of plants grown at low N, and the plants exhibited a low growth and low yield phenotype [41]. Therefore, overexpressing these glutamine synthetase genes unbalanced the C:N metabolic status in rice [41]. However, GS1 overexpression in wheat resulted in plants with increased total N in the grain and approximately 20% increase in grain yield [42]. Other transgenes, such as ENOD93 [52] and alaAT [12], showed increased levels of primary nitrogenous compounds and an NUE phenotype.

Previously, we suggested that manipulating regulatory pathways could provide a mechanism for improving NUE [55]. This hypothesis has been validated by He et al., who demonstrated that constitutive overexpression of miRNA826 and miR5090 repressed expression of glucosinolate synthesis-related genes while N starvation-responsive genes were upregulated [50]. Transgenic *Arabidopsis* expressing these miRNAs showed enhanced tolerance to low N, increased biomass, increased lateral root production, increased chlorophyll, and decreased anthocyanin content relative to the wildtype (WT) [50]. Likewise, overexpression of the *Zea mays* transcription factor Dof1 in rice caused increased phosphoenolpyruvate carboxylase (PEPc) expression as well as altered expression of TCA-related genes. Moreover, these expression changes were correlated with changes in TCA cycle intermediates, such as malate, citrate, and isocitrate [49]. Enhanced growth under limiting N, increased photosynthesis rate, and decreased shoot-to-root ratio was also observed [49]. Along these lines, overexpression of PEPc in *Vicia narbonensis* seedlings resulted in increased C and N content [56]. Increasing photosynthetic production by bioengineering crops is not only important for increased carbon sequestration [57] and biofuel production [58], but may also provide enhanced NUE as a result. Transgenic lines with increased photosynthetic capacity should therefore be evaluated in terms of nitrogen use efficiency as well, and vice-versa.

Several studies have suggested a link between photosynthate production (the saccharide products of carbon fixation) and NUE. Photosynthates are stored as a variety of polysaccharides including: starch, cellulose, hemicellulose, pectin, and lignin [59]. These polysaccharides are synthesized from nucleotide diphosphate-sugar (NDP-sugar) moieties such as UDP-α-d-glucose, which is a major component of cellulose, synthesized from fructose-6-phosphate, itself a product of photosynthesis [59]. Under N-limiting conditions, both *Arabidopsis* and rice show that genes encoding UDP-glucose 4-epimerases are differentially expressed [60,61], while the addition of N results in decreased expression of genes involved in cellulose biosynthesis [62]. Moreover, Guevara et al. recently showed that overexpression of the rice UDP-glucose 4-epimerase OsUGE1 led to increased sucrose and decreased cellulose production under nitrogen limiting conditions [63]. Similarly, overexpression of OsUGE1 in *Arabidopsis* resulted in drought, freezing, and salinity tolerance, which was attributed to elevated raffinose content [64]. This observation is consistent with a variety of studies that have reported similar phenotypes under N-limiting conditions (Table 1). Another study by Li et al. [65] demonstrated that transgenic *Arabidopsis* plants that overexpressed *Larix gmelinii* UDP-glucose pyrophosphorylase showed more rapid vegetative growth compared to wild-type plants, and had greater soluble sugar and cellulose levels [65]. An inverse relationship between the distribution of photosynthate into cell wall materials (such as lignins), and the nitrogen supply [66] suggests that there is an increase in carbon skeleton demand during nitrogen assimilation. This suggests that promoting carbon skeleton production for N assimilation through genetic manipulation may be an attractive means to enhance NUE. This diverse list of genes and gene products associated with the C:N balance in plants and hence biomass and yield shows the genetic complexity involved with improving NUE in plants. It is highly likely that a stacking transgenic approach, where two or more C:N metabolism-associated genes are coordinately over- or differently-expressed, would provide the correct metabolic balance for an NUE phenotype [8,9]. For example, when Wang et al. [43] co-expressed Dof1, GS1 and GS2 in tobacco, the transgenic plants had increased amino acids and sugars, decreased nitrate, malic acid and citric acid, and they exhibited a growth advantage over wild-type tobacco under low N conditions.

A wide variety of molecular genetic studies have demonstrated a significant association between carbohydrate metabolism and NUE. Moreover, these studies indicate that a more comprehensive understanding of plant metabolism could provide insight into directed approaches for engineering improved NUE. One of the major barriers to achieving this outcome is the difficulty in predicting the identity of genes that will have a positive impact on NUE metabolism. Metabolomics offers a promising solution for understanding the metabolic effects of genetic modification and possibly guiding NUE crop engineering.

### 2.3. Metabolomics Technology

The goal of metabolomics is to understand metabolic networks on a comprehensive scale by identifying and quantifying all of the metabolites present in biological extracts. This systems-level objective requires elements of traditional biology, analytical chemistry, computer science, and statistics. Although the relative weighting of these disciplines varies from study to study, most metabolomics investigations can be framed as follows: (1) a biologically relevant phenotype is identified; (2) metabolites are extracted from the relevant tissue(s); (3) observable metabolites are identified and quantified using a mixture of bioinformatics and bioanalytical approaches; and (4) metabolic phenomena associated with the biological phenotype are identified by statistical or computational analyses. The details of how to implement this strategy in plant-based metabolomics studies have been extensively reviewed elsewhere [67]. Herein, we will discuss the challenges and opportunities of metabolomics in NUE-related research.

One important consideration in adapting the emerging metabolomics technology to NUE is the significant role that analytical tools play in shaping experimental design and the impact these tools have on the nature and volume of data that are ultimately generated. Most metabolomics studies are conducted using nuclear magnetic resonance (NMR) spectroscopy or mass spectrometry (MS), as evidenced by PubMed title/abstract searches using the key words “metabolomics” or “metabolomics” and ”NMR” and “MS”.

**Figure 1:**
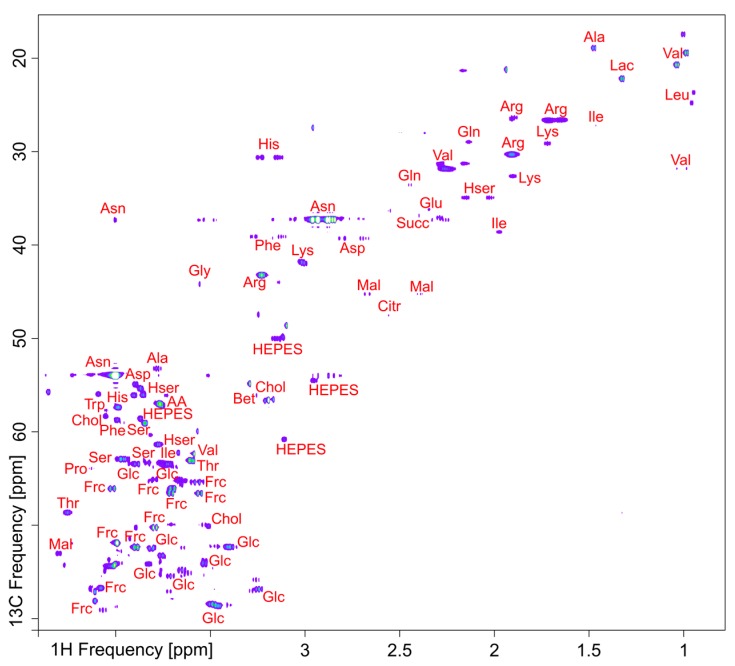
Multi-dimensional ^1^H-^13^C NMR spectrum of aqueous extracts from *Medicago sativa* seedlings illustrating a typical complement of metabolites observed in untargeted NMR.

The primary advantages of NMR are that its signals are directly proportional to concentration, and that it can detect virtually any molecule that is present above its sensitivity limit. These attributes are valuable in NUE studies because they allow one to account for all the carbon and nitrogen flowing into and out of systems, to investigate networks with no prior information about the organism’s metabolic architecture, and to unambiguously assign novel molecules. Moreover, NMR can detect ^13^C and ^15^N-containing molecules. Consequently, labeling studies employing these stable isotopes allows for the measurement of carbon and nitrogen utilization in plants [68]. Figure 1 shows a multidimensional ^1^H-^13^C NMR spectrum of *Medicago sativa* seedlings that illustrates a typical complement of metabolites observed in an untargeted NMR-based assay. Detailed descriptions on the method of collection and interpretation of these NMR data have been published elsewhere [69,70,71]. Additionally, in vivo NMR can be used to analyze metabolic pathway activity and metabolite compartmentalization in living plants [72,73,74]

**Figure 2:**
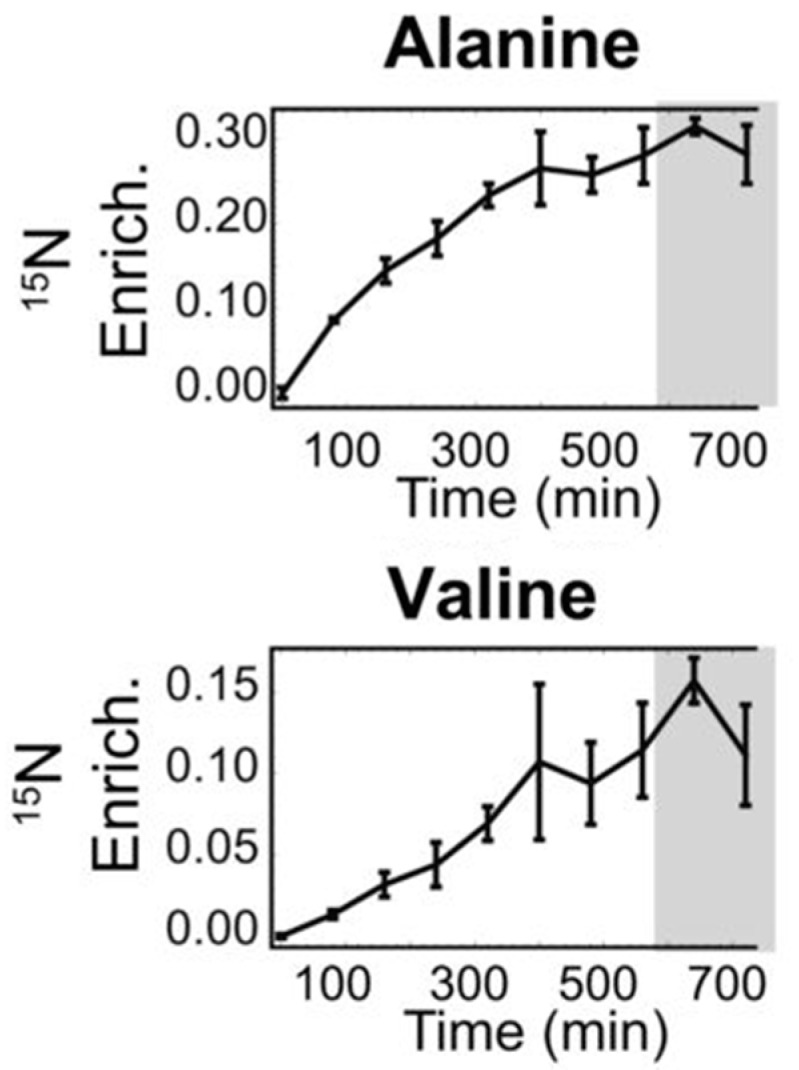
^15^N enrichment levels of two amino acids in barley leaves provided with ^15^N-labelled KNO_3_, as determined by gas chromatography-mass spectrometry (GC-MS). Light periods are marked in **white** and dark periods in **gray**. Adapted with permission from [78].

The primary advantage of mass spectrometry is its high sensitivity. Whereas NMR is restricted to analyses of the 20 to 50 most abundant compounds, MS can detect hundreds or even thousands of molecules per sample. This is invaluable for understanding metabolic networks on a comprehensive scale and can detect potent low-abundant compounds, such as hormones [75]. MS’s ability to investigate metabolic activity makes it a powerful tool for linking metabolism to genes. Recently, a variety of genes have been identified by coupling modern metabolomics methods to quantitative trait locus mapping (mQTL) [76]. This hybrid metabolomics/genomics approach offers a powerful mechanism for decoding the polygenic contributions to NUE phenotypes. Moreover, MS is inherently well-adapted to measuring isotope incorporation into metabolic intermediates. Consequently, MS is frequently used for quantitative analysis of pathway flux, elucidating metabolic network architecture, and determining metabolic partitioning through the network. While MS can provide data on overall isotope incorporation of (low-abundance) metabolites, NMR can be used to further refine this information (for the more abundant metabolites) by providing the molecular positions of the labels. This information can be used to unambiguously assign the pathways that were used to synthesize the compounds in questions [77,78,79]. The use of MS to trace isotope labelling is illustrated in Figure 2, which shows the accumulation of ^15^N-labelled amino acids in barley leaves after exposure to ^15^N-labelled KNO_3_. The rates at which ^15^N accumulates is dependent on the metabolic pathways used to synthesize the compounds and are thus a direct measure of pathway activity. This data can be used to trace the connectivity of complex metabolic networks.

### 2.4. Metabolic Flux Analysis

Metabolic data can be divided into two major categories: steady-state and flux. Steady-state data reflect metabolite concentrations, whereas fluxes indicate the rates at which metabolites are used and consumed. Nitrogen use efficiency is inherently related to flux rather than steady-state metabolism. The combination of NMR and MS offers a powerful opportunity to understand nitrogen metabolism from a flux perspective and to identify the most effective mechanisms for improving the nitrogen used by plants.

Significant research has been devoted to understanding metabolic flux in biological systems through computational analysis. Flux balance analysis (FBA) is one of the most powerful and convenient of these approaches [80]. In contrast to traditional enzyme kinetics, FBA does not require detailed information about enzyme properties, concentrations of intermediates, or protein levels. Consequently, FBA provides insight into network dynamics without relying on these difficult-to-obtain data. When coupled with FBA, metabolomics offers a powerful mechanism for constructing and refining models of complex systems.

**Figure 3:**
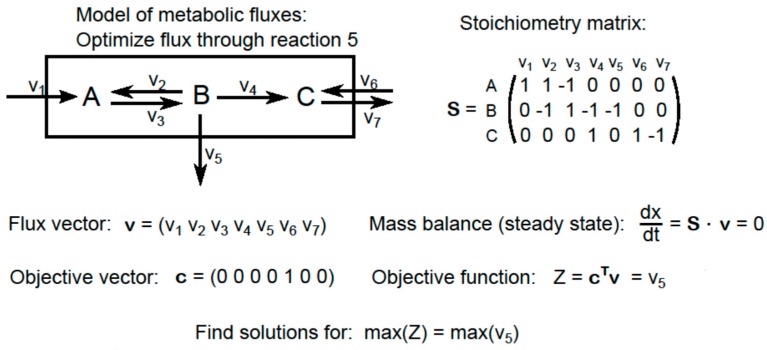
Example of a simple metabolic flux model and the equations for defining a flux balance analysis to maximize flux through reaction 5.

FBA analyses are performed by constructing a computational model of the metabolic network. This model includes a stoichiometry matrix, which describes all of the reactants and products involved in each reaction, and a flux vector representing all of the metabolic activity in the network. The goal of FBA is to find an optimal set of fluxes to achieve a particular objective [81] (see Figure 3 for an example). In the context of NUE, this may include maximizing biomass production with minimal N usage. Flux balance problems are generally solved by introducing constraints into the system, the most common of which is the steady-state assumption [79,80]. In addition, the system can be further constrained by establishing boundary fluxes, or the rates at which molecules enter and leave the system. Metabolomics is particularly useful in this context because it allows one to directly quantify boundary fluxes and to establish empirical constraints on metabolic partitioning within the network [82].

FBA has been used extensively to analyze nitrogen uptake in single cell systems such as *E. coli* [83]. However, analyses of whole plants are significantly more complex due to the significant differences in metabolism between organs, development stage, and complex environmental interactions. However, several groups have taken up this challenge, and FBA has now been used to elucidate the complex metabolic networks of plants and their symbionts [84,85,86,87]. A recent FBA study took a first step toward unraveling this complexity by incorporating two distinct types of plant cells (mesophyll and bundle sheath) and their interactions [88]. This approach has since been used to analyze nitrogen availability in the maize leaf [89]. Moreover, FBA has recently been performed using a whole-plant dynamic model of barley [25]. This model incorporated detailed flux maps of leaf, stem and seeds as well as simplified models of roots and phloem, and used further constraints predicted by functional plant models (FPM) [90].

In summary, recent studies have shown that plant metabolism can be understood through computational models. In general, these models have been reliant on computational predictions of metabolic constraints. Consequently, metabolomics offers a direct mechanism for advancing these state-of-the-art computational approaches. We anticipate that empirical measures of boundary fluxes and metabolic architecture defined by isotope-based studies will dramatically improve the quality and scope of these analyses.

### 2.5. Modeling Fluxes in NUE

Despite the recent developments in FBA of whole-plant metabolism, NUE has yet to become the focus of such efforts. Herein, we present a theoretical framework for a whole plant model of NUE and discuss the role that metabolomics could play in constructing this model.

Nitrogen use efficiency can be compartmentalized into nitrogen uptake efficiency (NUpE) and nitrogen utilization efficiency (NUtE). NUpE is the plant’s ability to take up fixed N from the soil and is both genetically and environmentally regulated. Plants can only use fixed, biologically reactive, nitrogen in N metabolism. Fixed N is available to plants as peptides and amino acids from decomposing organic matter and as NO_3_^−^ and NH_4_^+^ from soil microbes and synthetic and organic fertilizers. NUtE can be further defined as N assimilation and N remobilization. N assimilation is the plant’s ability to reduce nitrate to ammonia and use it as a substrate in the primary N metabolism reactions catalyzed by nitrate and nitrite reductases, glutamine synthetase and glutamate synthase. These reactions assimilate N into glutamine and glutamate, which are then used as N sources to produce other amino acids via amino transferases. These N compounds are translocated to the shoots and eventually remobilized and stored as N sinks in order to be a fixed N source for the embryo in the seed [8,91].

**Figure 4:**
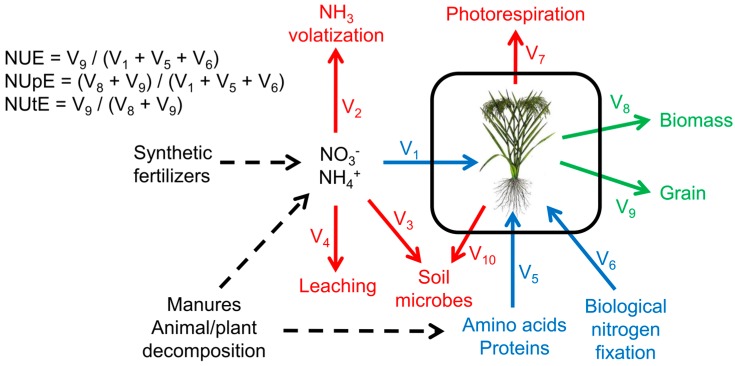
A simplified model of boundary fluxes in nitrogen use of crop plants. NUE: Nitrogen use efficiency, NUpE: Nitrogen uptake efficiency, NUtE: Nitrogen utilization efficiency.

The N inputs, the N flow though the plant, the N loss outputs and the N sinks can be measured as N boundary fluxes (V_1_ to V_9_ in Figure 4) using NMR techniques, and specific N metabolites can be traced through the system using MS techniques (Figure 2). Figure 4 shows the defined N fluxes for a field-grown cereal crop. The potential sources of N input are defined as V_1_, V_5_ and V_6_ (blue arrows), the potential sources of N loss (outputs) are V_2_, V_3_, V_4_, V_7_, and V_10_ (red arrows) and the N sinks are V_8_ and V_9_ (green arrows). N volatilization (V_2_) may be measured by means of headspace gas chromatography to further refine the model of boundary fluxes in the system. Together, these N flux data can provide valuable insight on the fate of N in the system, and identify flux changes when comparing wild-type and transgenic plants.

## 3. Conclusions

Recent advances in mass spectrometry and nuclear magnetic resonance spectroscopy have revealed the potential of using metabolomics to unravel metabolic networks in plants. Metabolomics studies on nitrogen deprivation have shown that the metabolic profiles of plants change dramatically in response to nitrogen availability. Moreover, genetically-engineered plants with modified NUE show elevated levels of nitrogen-containing molecules. These preliminary findings indicate that metabolomics could offer a powerful approach for understanding plant N physiology and assist in strategies aimed at engineering NUE in crops. New methods that integrate metabolomics and computational approaches, such as quantitative trait locus mapping and flux balance analysis, offer a powerful new strategy for investigating the role that individual genes play in phenotypes. While there are still no FBA studies reported that analyze NUE on a whole plant scale, recent analyses of other plant phenotypes by FBA have demonstrated the feasibility of this approach. By using this integrative approach, we believe that NUE can be understood at an unprecedented level of detail. These insights are of global importance because they may result in more effective management of crops, better nutrient fertilization practices, and may ultimately lead to a new generation of engineered plants that make better use of the available nitrogen.

## Figures and Tables

**Table 1:** Effect of nitrogen starvation on the metabolome of photosynthesizers.

Species	*Synechocystis* sp. PC 6803	*Chlamydomos reinhardtii*	*Chlamydomonas reinhardtii*	*Arabidopsis thaliana*		*Zea mays*		*Zea mays*	
N depletion condition: length of time and concentration shift	4 h; 5 to 0 mM NH_4_^+^	24 h; 7 to 0 mM NH_4_^+^	1, 2, 6 days; 7.48 to 0 mM NH_4_^+^	0, 2, 10 days; 6 to 0 mM NO_3_^−^)		Inbred lines A188, B73; 0.15 or 15 mM NO_3_^−^		Inbred line B73 0.1 or 10 mM NO_3_^−^	
Technique	CE-MS, LC-MS/MS	GC-TOF-MS	GC-MS	GC-MS, anion HPLC		GC-MS		GC-MS	
Reference	[32]	[33]	[34]	[35]		[26]		[11]	
Metabolite			1–2–6d	Shoot	Root	A188	B73	Veg.	Mat.
Amino Acids (percentages, %)									
Alanine	200	6	44–87–64	66–27	86–84	11–15	28–40	18	18
Arginine	48		129–97–65			14–51	97–82	26	
Asparagine	55	18		64–20	163–24	11–85	32–78	1	
Aspartate	21	63	21–17–14	44–27	48–32	28–52	45–163	4	
Cysteine		135				58–57	72–83		
Glutamate	88	18	21–72–57	68–54	82–29	27–50	57–62	12	
Glutamine	43	10	58–50–56				25–96	3	
Glycine	262	35	76–161–86	56–22	178–706	8–38	21–131	3	
Histidine	136	55							
Isoleucine	576	250	38–42–29	116–67	103–90	48–40	61–49	14	
Leucine	688	59	25–21–14	139–70	97–78	56–38	84–34		40
Lysine	124	42	28–62–58	84–37	106–72	77–84	198–91	26	54
Methionine	241	18	146–201–215						32
Phenylalanine	433	44	16–25–11	129–66	73–44	49–42	64–40	19	
Proline	198	34	71–49–95	72–23	73–38	40–57	70–65	8	
Serine	472	14	21–61–42	134–75	86–57	11–31	25–86	3	
Threonine	349	246	38–187–95	81–46	97–46	25–28	47–74	6	26
Tryptophan	83	495	8–24–6			88–53	74–52	62	
Tyrosine	1284	111	24–51–26	171–92	103–90	40–54	71–61	21	40
Valine	235	33	40–57–50	91–66	103–90	44–47	58–61	10	
Organic Acids (percentages, %)									
Aconitate	115			55–30	102–65			45	45
Benzoate		73	43–114–102	92–85	64–90				
Citrate	59	56	133–1478–1134	59–25	156–45				886
Erythonate				134–242	113–120			50	48
Fumarate	1015	16	33–125–95	442–397	94–67	41–78	51–68	52	135
Glycerate	141	58	40–48–46	172–67	1209–5301				
2-oxoglutarate	360	46	95–45–114	105–86	152–97	80–95	108–45		
Lactate			39–63–54	109–130	22–52				
Malate	876	26	34–87–82	113–97	993–461	34–20	23–23	61	
Maleate			28–56–148	157–182	84–85			900	
Oxaloacetate		94	1–36–1	160–83	125–192				
Pyruvate	334	25	21–27–100	81–75	71–106	75–50	81–34		11
Shikimate	168	40		103–77	160–53	170–216	131–312	26	
Succinate	398	81	97–216–167	178–346	114–67				
Threonate		39	96–131–111	99–132	267–238				
Alcohols and Sugars (percentages, %)									
Glycerol		77	1.6–0.9–0.5	100–93	64–70				
Inositol		13	30–52–84	97–74	177–258			67	
Fructose			312–202–97	462–218	687–277	44–34	29–31	11	
Galactose			17–7–7	343–487	208–225			30	15
Glucose			27–10–3	405–545	413–515	30–21	29–24	6	
Maltose		62		95–93	79–86			121	
Mannose				223–184	132–362			16	24
Raffinose	183			973–7981	198–313	275–268	270–159	333	
Sucrose				89–89	99–110				71
Xylose			67–195–231	119–149	271–357				
Phosphorylated Compounds (percentages, %)									
6-phosphogluconic acid	136	6	120–177–171						
Fructose-6P: Fru-6P	148	64	20–44–73	65–55	76–65	72–296	137–526	21	
Fructose-1,6-*bis*P	82	67							
Glucose-1-P	119	61							
Glucose-6-P	148	89	53–99–44	70–57	88–84	77–356	131–559	14	
Glycerate-3P	139	161	52–230–83					11	150
*myo*-inositol-P			108–83–71	148–77	66–70				
Phospho*enol-*pyruvate	104	19							
Ribulose-5P	127	99				69–174	100–333		
Nitrogenous Compounds (percentages, %)									
γ-aminobutyric acid	536		167–114–43	204–138	217–96	29–25	38–39	8	
Adenine	100	9	24–52–56						
Citrulline	23	23				11–73	23–138		
Hydroxylamine		139	114–81–72	59–8	19–34				
Ornithine	21	6	127–87–59			10–72	48–94		
Putrescine		9	11–13–8					12	9
Uracil		10	13–17–18						

**Table 2:** Effect of transgene expression on metabolite levels in transgenic plants.

Genetic Construct	Conditions	Technique	Core Metabolomic Results (Compared to WT)	References
N metabolism				
*Oryza sativa* *GS1;1* and *GS1;2* overexpressed in *Oryza sativa* cv. Zhonghua 11 under the control of the *CaMV 35S* promoter	Metabolic analysis done on tillering stage roots and shoots of plants growth with Low N and Moderate N	GC-TOF-MS	Low N: GS1;1 and GS1; 2 increased sugars, organic acids, free amino acids in shoots and decreased in roots. Moderate N: same results for both lines in shoots as for low N, in roots GS1;1 increased sugars, organic acids and free amino acids GS1;2 roots had decreased metabolites.	[41]
*Pisum sativum* *AS1* overexpressed in *Nicotiana tabacum* under the control of the *CaMV 35S* promoter	16 h light/8 h dark, 21 day old plants grown in sand, fertilized with Hoagland solution with 10 mM NO_3_^−^	HPLC	10–100 fold increased Asn. Decreased Gln, Asp. No change in Glu.	[46]
*Hordeum vulgare AlaAT* overexpressed in *Oryza sativa* under the control of the root-specific *OsANT1* promoter	14 h light/10 h dark, 45 day old plants grown hydroponically in 0.5, 2.0, and 5.0 mM NH_4_^+^	HPLC	Increased Gln, Glu, Asn, Asp, and Arg in roots and shoots.	[12]
N recycling/protein degradation/C:N balance				
*Mus musculus ODC* overexpressed in *Populus nigra* under the control of a 2X *CaMV 35S* promoter	Cell cultures grown in MS media	HPLC	Increased Ala, Thr, Val, Ile, and GABA. Decreased Gln, Glu, Orn, Arg, His, Ser, Gly, Cys, Phe, Trp, Asp, Lys, Leu, Met.	[53]
*Arabidopsis FUM2* overexpressed in *Arabidopsis* under the control of a 2X *CaMV 35S* promoter	8 h light/16 h dark, plants grown for 42 days with 1.25 mg (low) or 31.5 mg (high) inorganic nitrogen	GC-MS	Increased starch, *FUM2* knockouts reduced fumarate levels, varied amino acid levels according to light cycle.	[54]
Regulatory transgenes				
*Zea mays* *Dof1* expressed in *Arabidopsis* under the control of the *CaMV* 35S promoter; also expressed in potato	Constant light, plants grown on modified MS medium; low N = 1 mM NH_4_NO_3_/1 mM KNO_3_; high N = 10 mM NH_4_NO_3_/10 mM KNO_3_	Hitachi amino acid analyzer; enzymatic assay	Increased total [amino acid], NH_4_^+^ Decreased glucose, malate No change in sucrose, citrate, or 2-OG Similar to transgenic potato	[48]
*Zea mays* *Dof1* expressed in *Oryza sativa* under the control of the *CaMV* 35S promoter	14 h day/10 h night, hydroponic growth at 360 (high) or 90 µM (low) NH_4_^+^	CE-MS/MS	Increased concentration of some amino acids under high and low [N]	[49]
N-responsive transgenes				
*Oryza sativa ENOD93* expressed in *Oryza sativa* under the control of the *35S C4PDK* promoter	16 h day/8 h night for 4 weeks then 10 h day/14 h night for 1 week for flowering, soil growth at 1 mM (low), 5 mM (median) or 10 mM (high) nitrate	Biochemical assays	Increased total amino acids in OsENOD93-ox line roots in all N levels but more so under N stress. No increase in amino acid levels in shoots. Higher biomass in OsENOD93-ox.	[52]
Co-expressed N metabolism and Regulatory transgenes				
*Arabidopsis Dof1, GS1, GS2* expressed in tobacco under the control of the leaf specific *rbcS* promoter from tomato	Growth in perlite and low N nutrient solution for 60 and 90 days	RP-HPLC and biochemical assays	Transgenic tobacco co-expressing Dof1, GS1, GS2 had increased amino acids, glucose, sucrose and decreased nitrate, malic acid, citric acid and showed growth advantages	[43]
